# Outcomes of Surgery in Extra-thoracic Solitary Fibrous Tumors from a Tertiary Cancer Center in India

**DOI:** 10.1007/s13193-025-02316-6

**Published:** 2025-09-13

**Authors:** Vishnu Santhosh Menon, Shraddha Patkar, Tanvi Shah, Mufaddal Kazi, Prakash Nayak, Bharat Rekhi, Jifmi Jose Manjali, Prabhat Bhargava, Nehal Khanna, Siddhartha Laskar, Mahesh Goel

**Affiliations:** 1 Department of Surgical Oncology, Tata Memorial Hospital, Homi Bhabha National Institute, Mumbai, 400012 Maharashtra India; 2 Department of Surgical Oncology, Hepatobiliary Surgical Services, Tata Memorial Hospital, Homi Bhabha National Institute, Mumbai, 400012 Maharashtra India; 3 Department of Surgical Oncology, Colorectal Services, Tata Memorial Hospital, Homi Bhabha National Institute, Mumbai, 400012 Maharashtra India; 4 Bone and Soft Tissue Services, Department of Surgical Oncology, Tata Memorial Hospital, Homi Bhabha National Institute, Parel, Mumbai, Maharashtra India; 5 Department of Pathology, Tata Memorial Hospital, Homi Bhabha National Institute, Parel, Mumbai, Maharashtra India; 6 Department of Radiation Oncology, Tata Memorial Hospital, Homi Bhabha National Institute, Parel, Mumbai, Maharashtra India; 7 Department of Medical Oncology, Tata Memorial Hospital, Homi Bhabha National Institute, Parel, Mumbai, Maharashtra India; 8 Room Number 324, Gastrointestinal Services OPD, Third Floor, Homi Bhabha Block, Tata Memorial Hospital, Ernest Borges Road, Parel East, Mumbai, 400012 India

**Keywords:** SFT, Solitary fibrous tumors, Retroperitoneal, Sarcoma

## Introduction

Solitary fibrous tumors (SFT) are a rare class of mesenchymal tumors constituting about 2% of all soft tissue tumors (1, 2,3). SFTs show a tendency to arise from serosal membranes of body cavities with thoracic sites being the commonest. Extra-thoracic SFTs arise in deep soft tissues of the abdominal cavity, head and neck, orbit, and proximal extremities. They represent less than 6% of retroperitoneal sarcomas and have an annual incidence of 1 case per one million person per year (1). The World Health Organization classification of 2020 described SFTs as intermediate potential low metastatic risk mesenchymal tumors (4,5). Due to their rarity and lack of high-level evidence, there is no consensus regarding management. Margin negative surgical excision remains the treatment of choice but the complexity and spectrum varies due to varied location and presentation. The previously used terms *Benign* and *Malignant* SFTs are currently avoided, as even the benign SFTs do rarely have local and metastatic recurrences with incidence up to 45% (6,7). Some series report relapse rates as high as 10% even after 10 years (7,8). Several risk stratification models had been proposed to predict recurrence and survival for SFTs, with the modified Demicco model being the most widely reported (8,9). This study reports surgical outcomes and relapse patterns of extra thoracic SFTs. We examine the role of adjuvant radiation, tailoring of follow-up based on risk stratification and utility of salvage treatment in recurrent cases.

## Methods

A prospectively maintained institutional database across various disease management groups (DMGs) and the electronic medical records (EMR), between 1 st January 2008 and 31 st March 2024, were assessed to identify patients operated for extra-pleural SFTs. The 2020 WHO classification criteria was used as the diagnostic criteria to define SFT and all intrathoracic sites were excluded (5). As a standard institutional protocol, decisions regarding diagnosis, need for preoperative biopsy, operability and approach to the surgery were taken in a multidisciplinary tumor board of the respective DMGs. Clinical information including patient demographics, pathological characteristics, surgical details, postoperative outcomes, local and systemic relapses, radiotherapy details, chemotherapy course, and long-term survival outcomes of patients operated for SFTs was collected from the database and EMR. The extent of the disease was confirmed with preoperative imaging, operative notes, and histopathology reports. Descriptive statistics were used to analyses clinical, pathological, and radiological features and management protocols. Overall survival (OS) was calculated as the duration in months from the date of diagnosis until the date of death due to any cause or until the date of last follow-up (minimum 6 months post surgery). Disease-free survival (DFS) was calculated as the duration in months from the date of surgery until the date of local or systemic recurrence or death due to any cause or until the date of last follow-up (minimum 6 months post surgery). Risk assessment was done based on the modified Demicco model which includes four parameters which are age, size of tumor, mitosis rate, and necrosis percentage and has three tiers (low, intermediate and high) (9). Data was analyzed using IBM SPSS version 29 software (10). Kaplan–Meier survival curves were used to estimate survival and we tested for significance with a log rank test with a *p* value of less than or equal to 0.05 taken as significant.

## Results

### Cohort Characteristics (Table 1)

**Table 1:** Cohort characteristics

1. Age	Median	Range
	**54.5 years**	**25 to 75 years**
**2. Sex**	**Frequency**	**Percentage**
Male	14	63.6%
Females	8	36.4%
**3. Sites**		
***Abdomino-pelvic***	15	68.2%
Retroperitoneum	5	22.7%
Pelvis	7	31.8%
Mesenteric	1	4.5%
Liver	1	4.5%
***Other sites***	7	31.8%
Extremity	5	22.7%
Orbit	1	4.5%
Nape of neck	1	4.5%
**4. Size of tumor**		
0 to 5 cm	5	22.7%
5 to 10 cm	2	9.1%
10 to 15 cm	7	31.8%
More than 15 cm	8	36.4%
**5. Symptoms**		
*Abdomino-pelvic*		
Lump abdomen	4	18.2%
Lower urinary tract symptoms	4	18.2%
Scrotal swelling (varicocele)	1	4.5%
Abdominal pain	5	22.7%
Loss of weight	1	4.5%
*Other sites*		
Extremity swelling	5	22.7%
Head/neck swelling	2	9.1%
**6. Preoperative biopsy**		
***Yes***	16	72.7%
Concordant	10	45.4%
Discordant	1	4.5%
Inconclusive	5	22.7%
***Not done***	6	27.3%
**7. Status of tumor excision**		
R0 resection	18	81.8%
R1 resection	3	13.7%
R2 resection	1	4.5%

**Fig. 1:**
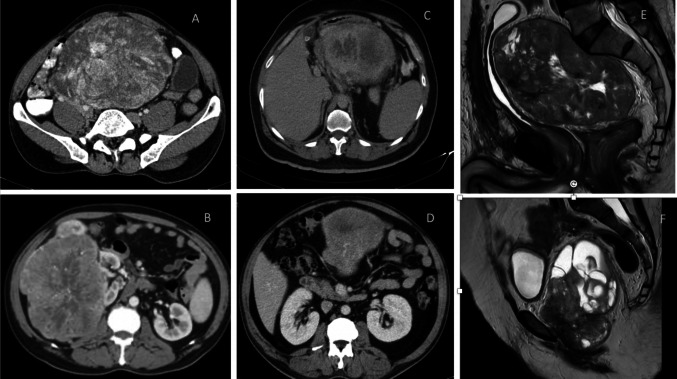
**A, B** Axial view of contrast enhanced CT scan of abdomen of right retroperitoneal SFTs. Both cases required right nephrectomy. **C** Axial view of contrast enhanced CT scan of abdomen of left sided SFT. This case required a Distal pancreatico-splenectomy. **D** Axial view of contrast CT scan of abdomen scan of mesenteric SFT. The superior mesenteric vessels are posterior and pancreas superior to the mass. **E** T2-weighed sagittal MRI images of a male patient with pelvic SFT. This case required Abdominoperineal resection as the tumor though well delineated was involving pelvic diaphragm. **F** T2-weighed sagittal MRI image of a female patient with pelvic SFT. This case was managed without multiorgan resection

**Fig. 2:**
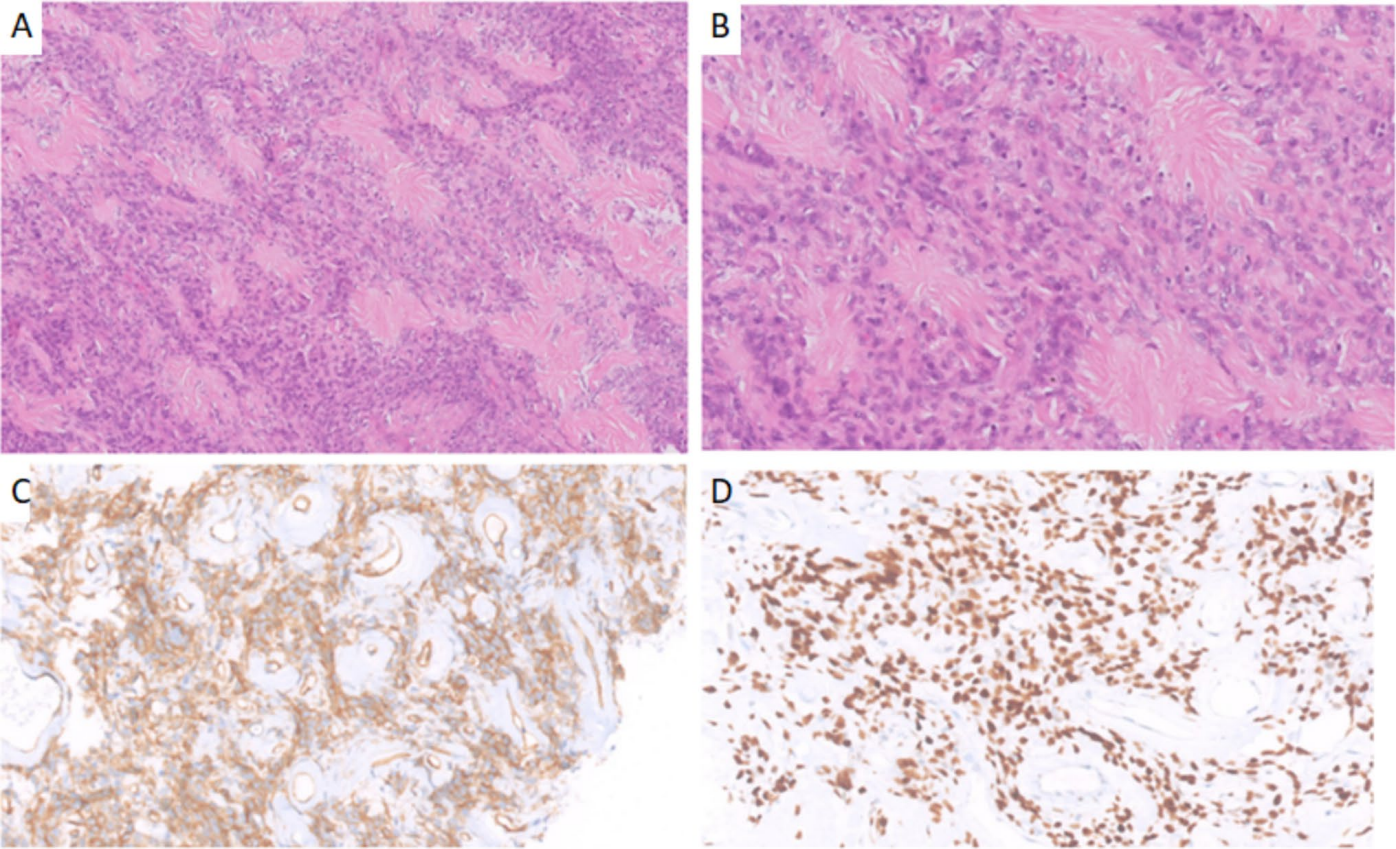
**A** Cellular tumor composed of spindle-shaped cells with intervening stromal collagen. Hematoxylin and Eosin slides, × 200. **B** Higher magnification showing relatively monomorphic oval to spindle-shaped cells with intervening thick collagen. **C** Tumor cells showing diffuse CD34 positivity, including intervening blood vessels. **D** Same case showing diffuse intranuclear STAT6 positivity

We identified 179 patients with SFT registered at our center for the said duration, of which 111 patients (62%) were extra-thoracic SFTs. Out of this 22 patients (19.8%) who had underwent surgical resection formed the cohort of patients for the study. The median age at presentation was 54 years (range 25 to 75 years). There were 14 male (63.6%) and 8 female (36.4%) patients which included 15 patients with abdominopelvic SFTs, 5 patients with extremity SFTs, and 2 patients with extra-meningeal head and neck SFTs. Patients were initially evaluated with a contrast-enhanced computed tomography (CECT) scan. SFTs are characterized as well-circumscribed soft tissue masses that enhance avidly indicating their vascular nature with smaller lesions being more homogenously enhancing and larger lesions having a central low attenuation area due to necrotic changes (Fig. 1). In extremities, pelvic and orbital SFTs, magnetic resonance imaging (MRI) was done and all cases had demonstrated the characteristic black-and-white-mixed pattern in T2-weighted sequences (Fig. 1). Radiological features although not diagnostic often helped to point towards the diagnosis (11). Preoperative biopsy was done in 16 patients. Surgical morbidity with the Clavien-Dindo Grade 3 or more complications were encountered in 2 patients (9.1%) with both these patients requiring re-exploration due to bowel related complications. Microscopically margin negative resection was achieved in 18 of patients (81.8%) and node positive disease was reported in 1 patient. The median size of tumor in the largest dimension was 12 cm (range: 3 to 30 cm). All cases were diagnosed by their characteristic appearance and CD34 and/or STAT6 expression (5). Immunohistochemical (IHC) markers for STAT6 were positive in all 8 out of 8 patients that were tested. IHC for CD34 was positive in 19 out of 22 in which it was done; BCL2 was positive in 7 out of 9 patients and S100 in 3 out of 17 patients in which they were done. NAB2-STAT6 fusion testing though gold standard was not performed in any patients due to logistic constraints (Fig. 2).

### Abdomino-pelvic SFT(Fig. 1)

This included 15 patients of which 8 were pelvic site primaries, 5 were retroperitoneal, 1 was mesenteric, and 1 was primary from the liver. Further, the pelvic site SFT also included 1 case of urinary bladder SFT and 1 prostatic SFT. The median age was 55 years ranging from 25 to 75 years, and 11 out of 15 cases were males. All patients were symptomatic on presentation with the most common symptom being abdominal pain. SFTs are known to be associated with paraneoplastic events including reactive hypoglycemia. In our cohort, only one patient had the same in the form of hypoglycemic episodes at initial presentation which resolved after resection (12). The median tumor size was 15 cm (range 4 to 33 cm). Intraoperatively, these tumors were usually found to have well-defined margins, with adjacent organs being pushed rather than invaded. Despite being circumscribed, multivisceral resections were required in 4 patients. Two patients with epicenter in the right retroperitoneum required nephrectomy due to involvement of the renal vasculature. Similarly, one patient required a distal-pancreatic-splenectomy from the invasion of the splenic hilum and tail of the pancreas (Fig. 1C). Another patient required an abdominoperineal resection as the disease involved pelvic diaphragm and anal canal (Fig. 1E). Rarer sites of abdominal primary which included the liver, prostate, and urinary bladder required appropriate organ-specific resections. A patient with prostate SFT who underwent radical prostatectomy had microscopic positive margin and thus was given adjuvant External Beam Radiotherapy (EBRT) to prostate bed and pelvis. Similarly, another patient with pelvic floor SFT who had margin positive tumor excision received postoperative EBRT. Neither of these two patients who had received postoperative radiotherapy for positive margins developed local or systemic relapses. There were no local recurrences and three patients had systemic relapse with a median Disease-Free Interval (DFI) of 54 months.

### Extremity SFT

There were five patients with a median age of presentation of 43 years (range 28 to 65 years) with a median size of 12 cm (range 8 to 18 cm) and of these four cases had epicenter in proximal thigh. Preoperative biopsy was done in all patients, and none of the patients received any neoadjuvant treatment. All underwent curative intent margin negative resections. One patient with planned close margins along bone required adjuvant radiation. There were no local recurrences, and one patient had systemic relapse in lung after a DFI of 27 months.

### Other Rarer Sites of SFT

For rare sites like orbit and neck, the focus remained on gross tumor excision and was achieved with acceptable morbidity. In the one patient with right sided orbital SFT, gross tumor resection was achievable but the same was removed piecemeal due to difficult access. In this patient, preservation of eye-ball was feasible without need for orbital reconstruction, as the orbital framework was left intact. EBRT was given to prevent local recurrence due to possible intraoperative tumor seeding. The second patient with upper paravertebral mass received postoperative radiotherapy due to planned close margins and ultimately microscopic positive margins at lamina. Neither of these two patients reported any local and systemic at a median DFI of 122 months.

### Survival Trends and Recurrence Patterns (Fig. 3, Table 2)

**Fig. 3:**
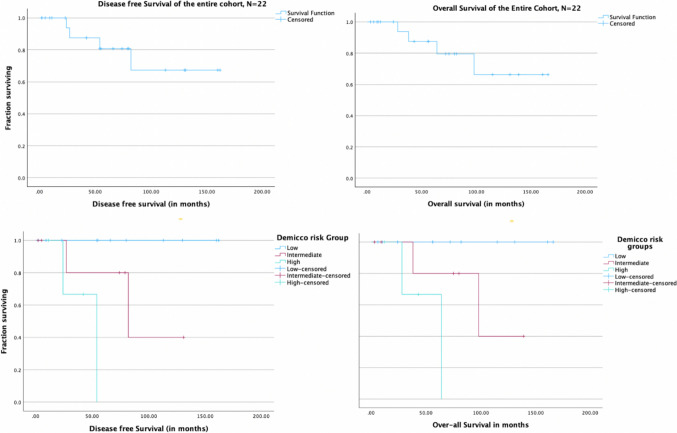
Kaplan–Meir survival curves of DFS and OS for the entire cohort and Demicco model risk groups

The median follow-up duration was 54 months (4 to 162 months). Four patients developed recurrences with three of them occurring after 50 months. Salvage treatment was attempted in all patients at the time of recurrence as they had good performance status, DFI more than 24 months and were single site recurrences. Salvage with systemic chemotherapy was attempted for three patients with lung metastasis with ifosfamide-adriamycin used as first line. Salvage radiation was offered to one patient with bone relapse. Median survival after recurrence was 10.5 months, and all patients who recurred died due to disease progression. The median DFS and OS were not reached for the cohort. The five-year DFS and OS were 80% and 80%, respectively.

**Table 2:** Factors determining disease-free survival and overall survival

Parameter		Overall, *N* (%)	Recurrences, *N* (%)	5-year DFS (%)	*p* value	OS events, *N* (%)	5-year OS (%)	*p* value
**Sex**	*Male*	14 (64)	4 (28.6)	72	0.171	4 (28.6)	72	0.181
	*Female*	8 (36)	0 (0)	100		0 (0)	100	
**Age**	*Less than 60*	15 (68)	2 (13.3)	90	0.36	2 (13.3)	90	0.388
	> *60 years*	7 (32)	2 (28.6)	56		2 (28.6)	56	
**Epicenter**	*Abdominopelvic*	15 (68)	3 (20)	80	0.784	3 (20)	80	0.786
	*Other sites*	7 (32)	1 (14)	80		1 (14)	80	
**Mitosis**	< *4*	16 (73)	1 (6.25)	91	0.056	1 (6.25)	91	0.071
	*4 or more*	6 (27)	3 (50)	53		3 (50)	53	
**Necrosis**	< *10*	17 (77)	2 (11.7)	80	0.267	2 (11.7)	81	0.249
	> *10*	5 (23)	2 (40)	75		2 (40)	75	
**Size**	*5 cm or less*	5 (23)	1 (20)	100*	0.075	1 (20)	100*	0.074
	*5–10 cm*	2 (9)	0(0)	100		0(0)	100	
	*10–15 cm*	7 (32)	0 (0)	100		0 (0)	100	
	*More than 15 cm*	8 (36)	3 (37.5)	50		3 (37.5)	50	
**Resection margin**	*Positive*	4 (18)	1 (25)	75	0.841	1 (25)	75	0.677
	*Negative*	18 (82)	3 (16.7)	83		3 (16.7)	84	
**Demicco risk group**	*Low*	11 (50)	0 (0)	100	0.033	0 (0)	100	0.044
	*Intermediate*	7 (32)	2 (28.6)	80		2 (28.6)	80	
	*High*	4 (18)	2 (50)	67		2 (50)	67	
**Postoperative radiotherapy**	Yes	5 (23)	0 (0)	100	0.084	0 (0)	100	0.084
	No	17 (77)	4 (23.5)	70		4 (23.5)	70	

*Recurrences happened after 5 years; hence, 5-year DFS was 100%

Out of 22 patients, 11 were low risk, 7 were intermediate risk, and 4 were high risk as per the Demicco model. The 5-year DFS and OS in the low-risk group were 100% and 100%, respectively; intermediate risk group 80% and 85%, respectively; and high-risk group 67% and 60% respectively (Fig. 3). Two patients each in intermediate risk and high risk Demicco groups developed recurrences. None of the low-risk Demicco patients developed recurrences.

## Discussion

SFTs are ubiquitous and represent a spectrum of mesenchymal fibroblastic tumors (13). Our cohort of patients demonstrated heterogeneity in presentation across varied organ systems and required a wide array of surgeries (Table 1). Hence, it was difficult to define a true denominator to describe the burden of this pathology in our clinical practice. Surgical resection with microscopic negative margins was achieved in majority of patients with acceptable peri-operative morbidity and excellent long-term survival (Table 2). Even though there is no strong evidence for any non-surgical treatment outside of the domain of extremity sarcoma either as an adjunct or in the setting of a recurrence as salvage measure, the possible role of radiation as a salvage tool needs to be explored further in cases with positive or close margins (14). In the context of extremity sarcoma, evidence for pre- or postoperative radiotherapy exists in the domain of limb salvage surgeries and often the same is extrapolated to other sites (15). The STRASS trial which compared role of preoperative radiotherapy did not show any benefit in abdominal recurrence-free survival when added to surgery (16). In further exploratory analysis of the data from the STRASS trial which also included SFTs, no benefit was seen in any subgroup other than well-differentiated liposarcoma. In our cohort, 3 out of 4 patients with margin-positive resection underwent postoperative radiotherapy and none of these three had relapses at a median follow up of 131 months. The one patient with R1 resection who did not receive adjuvant radiotherapy recurred after 50 months from surgery. A retrospective analysis from EuroNET group had reported reduced risk of local failures in patients with less favorable resection margins by combining radiation similar to what was observed in our cohort (17). Further, the role of chemotherapy and targeted therapy is not well-established in operable SFTs (18, 19).

Long-term survival after surgery remains feasible which highlights need for primary treatment in the form of surgery at centers experienced with sarcomas management (20). In the current study, 18% developed recurrences with three out four of them being initially R0 resections. All were late systemic relapses with no local site recurrences. Demicco et al. in 2019 compared various risk stratification models in SFT for their utility in predicting metastasis and overall survival (21). In our study, none of the patients with low risk in Demicco risk stratification had any recurrence while 50% of high risk and 28.5% intermediate risk had recurrences, similar to the risk predicted by the original study by Demicco et al. (8, 21). This may allow de-escalation of follow-up in low-risk patients while underscoring the need for long-term follow-up in high-risk groups. Potential utility of such risk stratification models for identifying patients who could benefit from adjuvant strategies like radiation needs to be studied further. Recurrent disease has poor outcomes with dismal response to salvage treatment modalities which is similar to the outcomes reported by Baldi et al. and O’Neill et al. (22, 23). Further research into the understanding the vascular signatures and patterns could be exploited in developing novel treatment options with use of anti-angiogenic therapy for such recurrent cases which otherwise have poor outcomes (19).

The major strength of the current study is that it is the single largest cohort of surgically treated extra-thoracic SFTs from a tertiary cancer center in South Asia, describes real world outcomes, and reflects our current clinical practices. The limitations of our study includes its retrospective nature, single institution data and smaller numbers. Further the smaller sample sizes make the statistical comparison between distinct subgroups within the study population limited and adjustments for same by statistically tools like Bonferroni correction would have lead to an overly conservative estimate of significance. Also while the study identifies potential utility of postoperative radiotherapy and the limited benefit of salvage treatment in recurrent setting, it will be ill-fitted to univocally conclude this based on our study alone due to the small study population and lack of proper control. However, through multi-institutional collaboration through the National Cancer Grid of India we hope to build a nation-wide database for such rare tumors which could serve in building better tools to improve our understanding and develop tailored management of such diseases (24).

## Conclusion

Surgical resection with negative microscopic margin is the standard of care of SFTs. Adjuvant radiotherapy can be used in margin positive resections to reduce chances of recurrence. Recurrences are usually late onset systemic failures, occurring in intermediate or high-risk Demicco group patients and have limited response to salvage therapy. Development of better adjuvant and salvage options remains an unmet need in high-risk patients.

## Data Availability

The data that support the findings of this study was completely anonymized and  can be made available on reasonable request from the corresponding author.
